# Complete Sequence of Succinamopine Ti-Plasmid pTiEU6 Reveals Its Evolutionary Relatedness with Nopaline-Type Ti-Plasmids

**DOI:** 10.1093/gbe/evz173

**Published:** 2019-08-06

**Authors:** Shuai Shao, G Paul H van Heusden, Paul J J Hooykaas

**Affiliations:** Molecular and Developmental Genetics, Institute of Biology, Leiden University, The Netherlands

**Keywords:** *Agrobacterium tumefaciens*, Ti-plasmid, succinamopine, nopaline, pTiEU6

## Abstract

*Agrobacterium tumefaciens* is the etiological agent of plant crown gall disease, which is induced by the delivery of a set of oncogenic genes into plant cells from its tumor-inducing (Ti) plasmid. Here we present the first complete sequence of a succinamopine-type Ti-plasmid. Plasmid pTiEU6 is comprised of 176,375 bp with an overall GC content of 56.1% and 195 putative protein-coding sequences could be identified. This Ti-plasmid is most closely related to nopaline-type Ti-plasmids. It contains a single T-region which is somewhat smaller than that of the nopaline-type Ti-plasmids and in which the gene for nopaline synthesis is replaced by a gene (*sus*) for succinamopine synthesis. Also in pTiEU6 the nopaline catabolic genes are replaced by genes for succinamopine catabolism. In order to trace the evolutionary origin of pTiEU6, we sequenced six nopaline Ti-plasmids to enlarge the scope for comparison to this class of plasmids. Average nucleotide identity analysis revealed that pTiEU6 was most closely related to nopaline Ti-plasmids pTiT37 and pTiSAKURA. In line with this traces of several transposable elements were present in all the nopaline Ti plasmids and in pTiEU6, but one specific transposable element insertion, that of a copy of IS1182, was present at the same site only in pTiEU6, pTiT37, and pTiSAKURA, but not in the other Ti plasmids. This suggests that pTiEU6 evolved after diversification of nopaline Ti-plasmids by DNA recombination between a pTiT37-like nopaline Ti-plasmid and another plasmid, thus introducing amongst others new catabolic genes matching a new opine synthase gene for succinamopine synthesis.

## Introduction

*Agrobacterium tumefaciens*, a Gram-negative plant pathogen belonging to the family *Rhizobiaceae*, is the causative agent of crown gall disease, which causes severe losses in agriculture. It induces tumor formation in plants by transferring a segment of its tumor-inducing (Ti) plasmid, the T-DNA, to plant cells, and has been developed as a vector to create transgenic plants and fungi and *Agrobacterium*-mediated transformation has become the preferred method of transformation of these organisms over the past decades. 

In the tumors induced by *A. tumefaciens*, tumor-specific metabolites called opines are formed under control of genes present on the T-DNA. According to the different opines found in the tumors, Ti-plasmids are classified as nopaline-type, octopine-type, agropine-type, vitopine-type, succinamopine-type, and chrysopine-type. Examples of most of these Ti-plasmid types have now been characterized in detail by DNA sequencing. The octopine Ti-plasmid (Zhu et al. 2000) and the nopaline Ti-plasmid pTiSAKURA (Suzuki et al. 2000) were the first that were fully characterized by DNA sequencing. Later with the advent of next-generation sequencing sequences of several other Ti-plasmids including nopaline-type pTiC58 (Goodner et al. 2001; Wood et al. 2001), octopine-type pTiAch5 (Henkel et al. 2014; Huang et al. 2015), vitopine-type pTiS4 (Slater et al. 2009), agropine-type pTiBo542 (Oger et al. 2001), and chrysopine-type pTiChry5 (Shao et al. 2018) became available. Here, we present the first sequence of a succinamopine-type Ti-plasmid, that of pTiEU6. Previously, it was shown that succinamopine strains such as EU6, AT181, and T10/73 contain a Ti plasmid which has a restriction profile that resembles that of nopaline Ti plasmid pTiT37 to some extent (Sciaky et al. 1978). These strains induce tumors in which succinamopine/asparaginopine (SAP), succinamopine lactam (SAL), and succinopine lactam (SOL) are present, but not nopaline (Chang and Chen 1983; Chilton et al. 1984). Succinamopine is a conjugate of α-ketoglutaric acid and asparagine, which is structurally related to nopaline, which is a conjugate of α-ketoglutaric acid and arginine. Although it was originally reported that these strains could degrade nopaline (Montoya et al. 1977), later it was reported that this was not the case, but that they could degrade leucinopine, a conjugate of α-ketoglutaric acid and leucine, besides SAP and SOL (Chilton 1985a). Agropine strains such as Bo542 and chrysopine strains such as Chry5 produce tumors that besides agropine and chrysopine, respectively, also contain leucinopine and succinamopine, which they also can degrade. However, leucinopine and succinamopine produced in tumors induced by Bo542 and Chry5 have the unusual l,l-stereochemistry. Instead the d,l-isomer of leucinopine and succinamopine is made in tumors induced by strains carrying pTiEU6 (Chilton 1985a, 1985b). By comparing the pTiEU6 sequence to that of a set of nopaline Ti-plasmids, including six newly sequenced nopaline Ti-plasmids, we found that pTiEU6 lacks the nopaline synthase gene in the T-region and the adjacent genes for nopaline import and catabolism, but instead has obtained a gene for succinamopine synthase in the T-region and an adjacent set of genes, which show similarity to the pTiBo542 and pTiChry5 genes involved in succinamopine and leucinopine import and catabolism. We conclude that pTiEU6 is derived from a nopaline Ti-plasmid related to the group of pTiT37 and pTiSAKURA by DNA recombination with another so far unknown plasmid.

## Materials and Methods

### Bacterial Growth and DNA Extraction

*Agrobacterium* *tumefaciens* strains LBA941 and LBA942, are Ti-plasmid-cured derivatives of strain C58, containing pTiEU6 and pTiKerr27, respectively, and were obtained as strains A289 and A293, respectively, from Dr E. W. Nester (Seattle, United States). Ti-plasmid pTiT37 is harbored in a derivative of strain T37, LBA8374 which is from our own strain collection. Wild-type strain LBA9670 was obtained as strain 1 from Dr S. Süle (Budapest, Hungary); strain LBA9280 was obtained from Dr A. Kerr as Kerr108 and strains LBA8670 and LBA8790 were received from Dr X. Nesme (Lyon, France) as strains CFBP2178 and CFBP1935, respectively. All *A. tumefaciens* strains were cultured in LC medium (Hooykaas et al. 1977) with appropriate antibiotics at 29 °C.

After growth on LC medium at 29 °C, genomic DNA was isolated using the DNeasy Blood & Tissue Kit (QIAGEN) with a few modifications. Briefly, after the cells were harvested, the pellets were re-suspended in TES buffer (0.01 M Tris–HCl pH 8.0, 0.05 M NaCl, 0.05 M EDTA). Then the cells were treated with 400 μg/ml lysozyme for 30 min on ice. After these steps, the kit protocol was followed.

### DNA Sequencing, Sequence Assembly, and Analysis

Total genomic DNA was isolated from *A. tumefaciens* strains and 2 libraries were constructed using the HiSeq SBS kit v4. Paired-end 125 cycles sequence reads were generated using the Illumina HiSeq2500 system by BaseClear (Leiden, The Netherlands). After quality analysis, paired-end reads were retained with an average length of 125 bp. Initially, assembly was carried out using CLC Genomics Workbench software (version 7.0.4). Subsequently, the Blast 2.2.31+ algorithm (NCBI) was used to map all contigs to the reference sequences, nopaline-type pTiC58 (AE007871) (Wood et al. 2001) and octopine-type pTiAch5 (CP007228) (Henkel et al. 2014). Based on sequence conservation among Ti-plasmids and topology, possible contigs with high hits were identified. The relative order and relationship of these contigs was determined by a series of PCRs and gaps were filled in by sequencing relevant positive PCR products. PCR products were purified by agarose gel electrophoresis and sequenced by Macrogen (Amsterdam, The Netherlands) and all primers were obtained from Sigma-Aldrich.

Protein-coding genes were predicted using the program Glimmer (version 3.02) (Delcher et al. 2007) and submitted to the RAST annotation system (http://rast.nmpdr.org/) for functional annotation (Overbeek et al. 2014). The assembled sequence was annotated using the IGS Prokaryotic Annotation Pipeline as well (Galens et al. 2011). Functional characterization was generated using eggNOG-Mapper which employed the functional categories in COG, KOG, and arCOG databases (Huerta-Cepas et al. 2017). Insertion elements (IS elements), transfer-RNA (tRNA), and ribosomal RNA (rRNA) were identified using IS finder (https://www-is.biotoul.fr/), tRNAscan-SE (http://lowelab.ucsc.edu/tRNAscan-SE/), and RNAmmer (http://www.cbs.dtu.dk/services/RNAmmer/), respectively. IslandViewer was used to predict genome islands (GI) (Dhillon et al. 2015) and CGView was used to generate circular maps of Ti plasmids (Stothard and Wishart 2005). Average nucleic acids identity (ANI) values between the query plasmid and the reference plasmid were calculated by the Perl script (Konstantinidis and Tiedje 2005). Comparative maps of complete Ti-plasmid sequences were constructed by CGView using BlastN (Stothard and Wishart 2005) and the Mauve program (Darling et al. 2010). The alignments were visualized by the web-tool Kablammo (Wintersinger and Wasmuth 2015) and sequence similarity of T-DNA borders was illustrated using Weblogo (http://weblogo.berkeley.edu/).

### Phylogenetic Analysis

Phylogenetic analysis was performed using MEGA version 7.0 (Kumar et al. 2016). Distances (distance options according to the Kimura two-parameter model) and clustering using the neighbor-joining (NJ) and maximum likelihood (ML) methods were determined using bootstrap values based on 1,000 replicates. The consensus trees represent combined data from three independent runs, with 100% consensus required for inference of relatedness.

The kSNP3 program (Gardner et al. 2015) was performed to identify core single nucleotide polymorphisms (SNPs) in the set of pTiEU6 and nopaline Ti-plasmids and to estimate phylogenetic tree based upon those SNPs. The optimal value of K-mer size was obtained by Kchooser of the kSNP3 package. The kSNP3 was conducted using the default parameters with the core and the ML tree options. After that a phylogenetic maximum-likelihood tree was generated using FigTree v1.4.3 software (Rambaut 2018). The branch lengths are expressed in terms of changes per number of SNPs.

## Results

### Genomic Overview of Succinamopine Ti-Plasmid pTiEU6

**Fig. 1.**
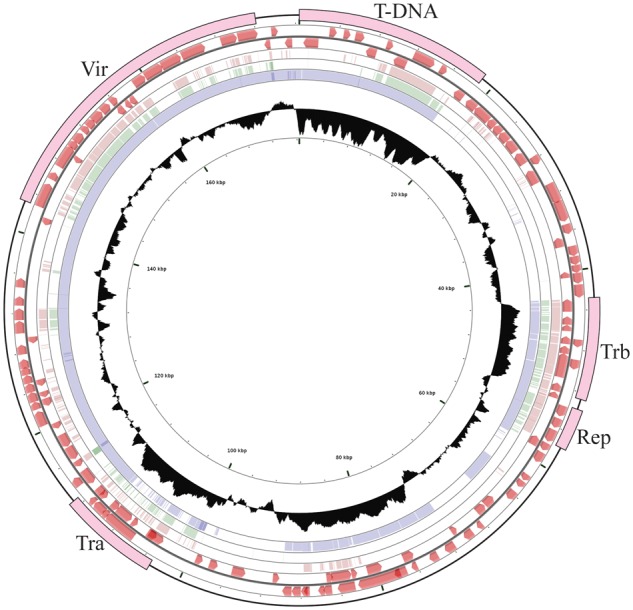
—Schematic circular map of Ti-plasmid pTiEU6. Circle ranges from 1 (outer circle) to 6 (inner circle). Circle 1, location of T-DNA, *tra*, *trb*, *rep*, and *vir* operons; Circles 2 and 3, predicted open reading frames on the plus strand and minus strand, respectively; Circles 4, 5, and 6, coordinated mapping of Ti-plasmids pTiBo542, pTiAch5, and pTiC58 against reference pTiEU6, respectively; Circle 7, G + C content percentages, median line represents the average GC content of the entire sequence. Single nucleotide based identities were expressed as height of the circles from blank to filled to represent 0-100% identity.

The complete sequence of pTiEU6 comprises 176,375 bp and has an average GC content of 56.1%. In total, 195 open reading frames (ORF) were identified with an average size of 761 bp (table 1 and fig. 1). To analyze the functional content of ORFs of pTiEU6, COG functional analysis was performed by eggNOG-Mapper. In total, 120 ORFs were assigned to COG categories, whereby the intracellular trafficking and secretion (group U) and unknown function (group S) categories contained the highest numbers of proteins (supplementary table S1, Supplementary Material online). The pTiEU6 plasmid carries one T-region of 18,789 bp with a GC content of 45.7%, which is significantly lower than other regions of the Ti-plasmid. Genes encoding tRNA or rRNA and GI could not be identified.

**Table 1:** General Features of Ti-Plasmid pTiEU6 and Newly Sequenced Nopaline Ti-Plasmids

Feature	pTiEU6	pTiT37	pTiKerr27	pTiSule1	pTiKerr108	pTiCFBP1935	pTiCFBP2178
Size (bp)	176,375	203,781	243,905	217,820	220,307	213,092	217,821
G+C (%)	56.1	55.9	57.2	56.8	56.8	56.8	56.8
Open reading frames (ORFs)	195	198	233	202	207	196	202
ORFs assigned into COG	120 (61.5%)	138 (69.7%)	167 (71.7%)	145 (71.8)	148 (71.5%)	142 (72.4%)	146 (72.3%)
T-region (bp)	18,789	23,415	24,835	24,477	24,477	24,477	24,477
G+C (%) of T-regions	45.7	45.8	46.1	45.8	45.8	45.8	45.8
IS elements	4	4	8	4	4	4	4

### Genetic Relatedness of pTiEU6 with Other Ti-Plasmids

**Fig. 2.**
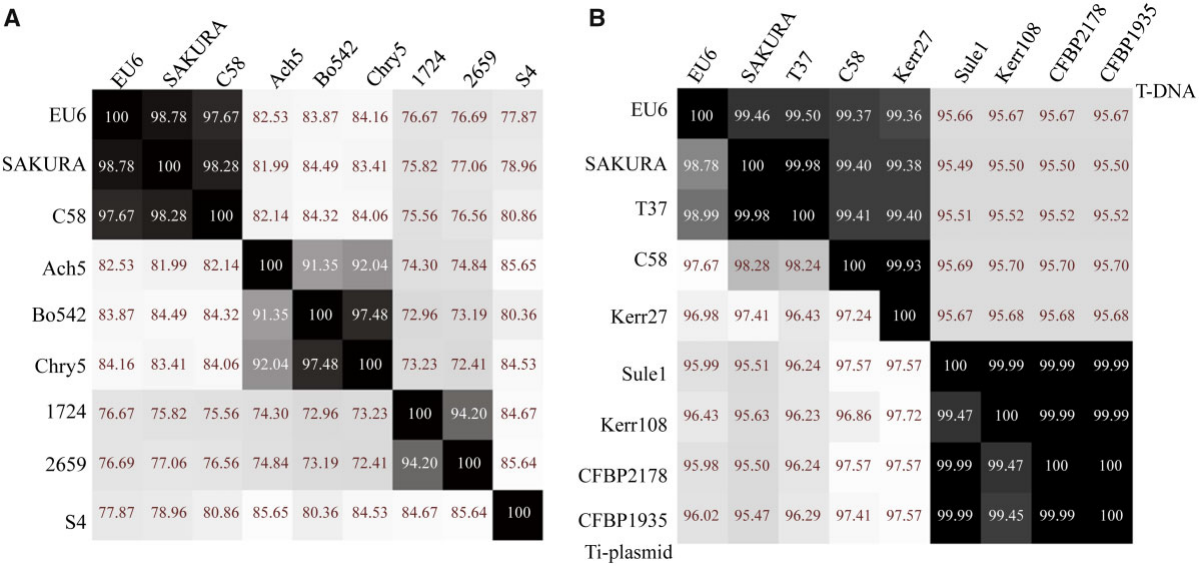
—Heatmap of average nucleotide identity (ANI) values. The ANI values between the query sequence and the reference sequence were calculated by the Perl script (Konstantinidis and Tiedje 2005) and visualized by Heatmapper (Babicki et al., 2016). (*A*) The ANI values of representative Ti-plasmids. (*B*) The ANI values of pTiEU6 and sequenced nopaline Ti-plasmids. The ANI values of T-DNA were shown in the upper right part and those of whole Ti-plasmids were present in the lower left part.

An ANI analysis was performed to determine the genetic relatedness of pTiEU6 with other Ti- and Ri-plasmids. To this end, we extracted the complete sequences of nopaline pTiC58 (AE007871; Wood et al. 2001) and pTiSAKURA (AB016260; Suzuki et al. 2000), octopine pTiAch5 (CP007228; Henkel et al. 2014), agropine pTiBo542 (DQ058764; Oger et al. 2001), chrysopine pTiChry5 (KX388536; Shao et al. 2018), mikimopine pRi1724 (AP002086; Moriguchi et al. 2001), cucumopine pRi2659 (EU186381; Mankin et al. 2007), and vitopine pTiS4 (CP000637; Slater et al. 2009) from public databases and calculated ANI values between pTiEU6 and them. As demonstrated in figure 2*A*, pTiEU6 shared the highest ANI values with nopaline Ti-plasmids pTiSAKURA (98.78%) and pTiC58 (97.67%), whereas ANI values of pTiEU6 were <85% with other opine type Ti- and Ri-plasmids. In addition, it became apparent that Ti-plasmids can be divided into two groups basically, which are nopaline and succinamopine Ti-plasmids on the one hand versus agropine, chrysopine, and octopine Ti-plasmids on the other hand. Pair-wise alignments of pTiC58, pTiAch5, and pTiBo542 with reference pTiEU6 made with the BlastN algorithm (fig. 1) confirmed that pTiEU6 shared most of the conserved regions with pTiC58 except for the opine catabolism region between T-DNA and *trb* operon. These results demonstrate that pTiEU6 is most related to the nopaline Ti-plasmids.

**Fig. 3.**
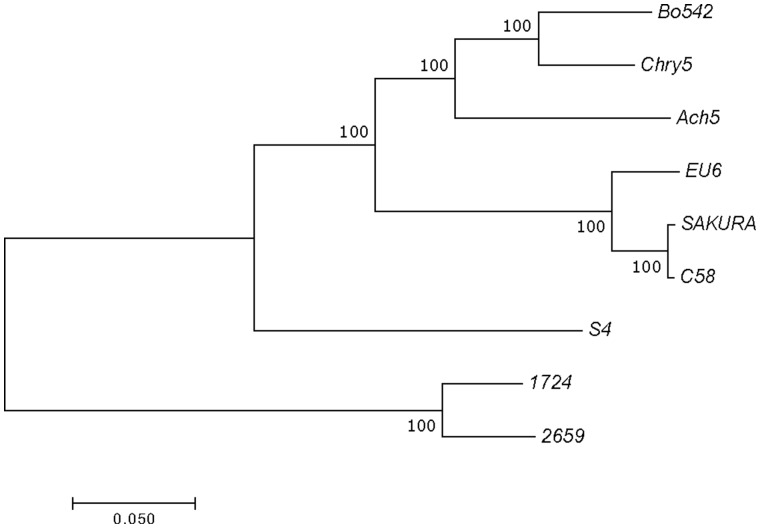
—Phylogenetic tree constructed from alignments for concatenated nucleotide sequences of the genes *virD2*, *repC*, *traR/M*, and *trbE* using the MEGA7.0 software. The topological structures of each tree are built and confirmed by ML and NJ methods. Bootstrap values from 1,000 replicates are located next to the branches and the evolutionary distances were computed with the Poisson correction. The scale bar represents the nucleotide substitutions rate units.

To confirm this evolutionary relationship at the gene level, a phylogenetic tree was constructed using the concatenated nucleotide sequences of the genes *virD2*, *repC*, *traR/M*, and *trbE*, which are involved in virulence, replication, regulation, and conjugation, respectively (fig. 3). In the tree, besides the corresponding genes from nopaline pTiC58, octopine pTiAch5, agropine pTiBo542, chrysopine pTiChry5, and vitopine pTiS4, the corresponding genes from two hairy root inducing plasmids, that is, mikimopine pRi1724 and cucumopine pRi2659 were included as well. In line with the results from ANI analyses, pTiEU6 was clustered with nopaline-type Ti-plasmids, which form an individual branch. The trees confirm the close relatedness of pTiEU6 with the nopaline-type Ti-plasmids.

### Evolutionary Relationship between pTiEU6 and the Class of Nopaline Ti-Plasmids

In order to get a more detailed insight into the evolutionary relationship between pTiEU6 and the nopaline Ti-plasmids, we went on to sequence six other nopaline Ti-plasmids from various geographical origins. The general features of these plasmids (pTiCFBP1935, pTiCFBP2178, pTiKerr27, pTiKerr108, pTiSule1, and pTiT37) are described in table 1, which shows that they have similar features and their sizes range from 203,781 bp up to 243,905 bp with a similar GC content of ∼56–57%. In addition, the predicted ORFs encoded by them present similar functional COG profiles (supplementary table S1, Supplementary Materials online).

**Fig. 4.**
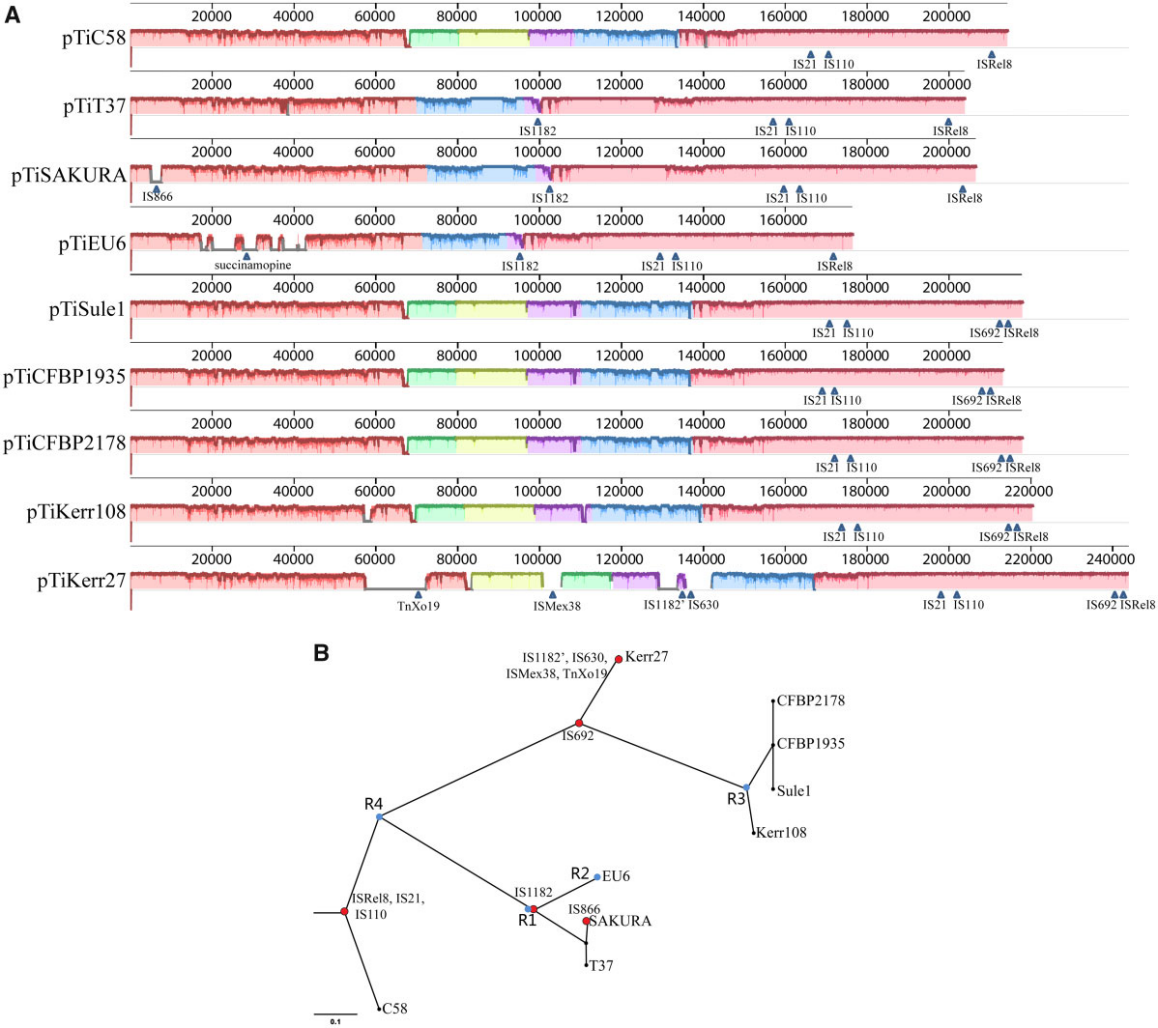
—(*A*) Schematic comparison of pTiEU6 and all sequenced nopaline Ti-plasmids. ProgressiveMauve (Darling et al., 2010) was used to generate pairwise alignments with default parameters and the single nucleotide based similarities were expressed as height of the panels from blank to filled to represent 0-100% similarity. Different colored blocks represent conserved regions and arrows indicate the site of transposons. (*B*) Core SNPs analysis. SNP-based phylogenetic tree of pTiEU6 and nopaline Ti-plasmids was visualized using FigTree v1.4.3 software (Rambaut, 2018). Core SNPs were identified by the kSNP3.0 program (Gardner et al., 2015). Red nodes represent the insertion of transposable elements. Blue nodes R1, R2, R3, and R4 represent DNA recombination events leading to exchanges in the area between *rep* and *tra* operon (R1), opine synthesis and catabolism area (R2), the area containing genes *6a* and *6b* (R3), and the area of *tra* operon containing genes *traG/D/C/A* (R4). The R4 event followed the insertion of three transposable elements ISRel8, IS21 and IS110, which are present in all nopaline Ti-plasmids. Later the R2 event took place after the acquirement of IS1182. Whether the recombination events R1 and R3 coincided or not in line with the acquirement of IS1182 and IS692, respectively, cannot be deduced from the data. The scale bar represents the nucleotide substitutions rate units.

The ANI values between pTiEU6 and all available nopaline Ti-plasmids were then calculated. As can be seen from figure 2*B* (lower left part), pTiEU6 shares the strongest similarities with pTiSAKURA (98.78%) and pTiT37 (98.99%). Similarities with the other nopaline Ti-plasmids are lower (95.98–97.67%). Similar results were obtained by ANI analyses of the T-regions (upper right part). Subsequently, global plasmid-wide sequence alignments were carried out, which are presented as linear maps by ProgressMauve (fig. 4*A*;supplementary fig. S1, Supplementary Materials online). The single nucleotide-based similarities were displayed as height of the panels from blank to full to represent 0–100% similarity and color frames indicate the sequences in these regions are conserved. Another phylogenetic relationship tree was deduced using SNP-based core sequence variations by the kSNP3 program (fig. 4*B*) (Gardner et al. 2015). The optimal value of K-mer size was 17 and obtained by Kchooser of the kSNP3 package. The number of core SNPs was 1,091 and the total number detected was 4,079. Using MUMmer, the numbers of SNPs were determined for each pair of Ti-plasmids (Kurtz et al. 2004). Compared with pTiEU6, the number of SNPs in nopaline Ti-plasmids were 1,556 (pTiSAKURA), 1,541 (pTiT37), 3,745 (pTiC58), 4,422 (pTiKerr27), 4,764 (pTiKerr108), 4,798 (pTiSule1), 4,831 (pTiCFBP1935), and 4,797 (pTiCFBP2178), respectively. All these data show that pTiEU6 is most similar to pTiSAKURA and pTiT37. We shall refer to these latter two plasmids as forming subgroup A of the nopaline Ti plasmids, the other studied nopaline Ti-plasmids forming subgroup B (pTiKerr27, pTiKerr108, pTiSule1, pTiCFBP1935, and pTiCFBP2178).

### Transposable Elements

Over time transposable elements may insert in chromosomes and plasmids and their presence at an identical position in different Ti-plasmids may thus point to a common descent. Several (remnants of) transposable elements were found in pTiEU6 and the nopaline type Ti-plasmids, which hereinafter will be referred to by the name of the most closely related element found in the IS finder database. However, this may reflect only limited similarity to these elements in short segments only. Nevertheless these turned out to be very useful in tracing the evolutionary relationships between these Ti plasmids. Transposable element ISRel8 (AgrTiEU6_207, IS66 family), IS21 (AgrTiEU6_149), and IS110 (AgrTiEU6_154) are present at an identical position in all nopaline Ti-plasmids and in pTiEU6. They apparently integrated before the diversification of these plasmids and their spread over the world. The insertion element IS1182 (AgrTiEU6_104) is present at an identical position only in the subgroup A nopaline Ti-plasmids and in pTiEU6. Apparently, this IS1182 copy was integrated in a group A nopaline plasmid before it recombined with another plasmid to generate pTiEU6. Plasmid pTiKerr27 also contains a copy of IS1182, in figure 4 called IS1182′, but at a completely different position and thus representing an independent insertion event. Another insertion element IS692 (IS66 family), occurs at a site close to ISRel8 in the subgroup B nopaline Ti-plasmids, except pTiC58. An insertion of IS866 is present specifically in the T-DNA of pTiSAKURA and must represent a relatively recent insertion event. A similar IS866 element was previously found to inactivate the *iaaH* gene in the T-DNA of several octopine Ti-plasmids (Paulus et al. 1989).

Plasmid pTiKerr27 is the largest nopaline Ti-plasmid discovered so far. This plasmid is a group B nopaline Ti plasmid, but it contains four additional transposable elements in areas with segments of novel genes. These areas may therefore have been introduced into pTiKerr27 by transposition. A gene cluster was found with partial similarity to the transposon module TnXo19 with genes *tniA/B/Q* (AgrTiKerr27_65/66/67) (Mindlin et al. 2001). Another transposable element related to ISMex38 (AgrTiKerr27_00095) is present in another nonhomologous region of pTiKerr27. Finally, a large set of nonconserved hypothetical genes was introduced along with insertion element IS630 (AgrTiKerr27_00128), which is located closely to IS1182′ (AgrTiKerr27_00126). Overall the evolutionary path revealed by the (remnants of) transposable elements thus concurs with the analyses by similarity scores of the whole Ti-plasmid and their individual genes.

### Regions Shared between pTiEU6 and Nopaline Ti Plasmids

As stated in the previous paragraph, we compared the main gene clusters at the nucleic acid level to determine the evolutionary relationship between pTiEU6 and nopaline Ti-plasmids by using ProgressMauve (fig. 4*A*). Taken altogether, the homologies are very high with the subgroup A nopaline Ti-plasmids in all areas except for the region responsible for nopaline and succinamopine catabolism, respectively. A large part of the region between *repABC* and the *tra* operon of almost 30 kb is strongly different between the subgroup A and subgroup B nopaline Ti-plasmids, as can be seen by the colored blocks in figure 4*A*. In pTiKerr27 the insertion of transposable element ISMex38 (AgrTiKerr27_00095) in the middle of this region has led to a local inversion, as shown in figure 4*A*, the green and yellow blocks. These results are again in line with results from SNPs and ANI analyses showing that pTiEU6 is more closely related to pTiSAKURA and pTiT37 than to other nopaline Ti-plasmids.

**FIG. 5.**
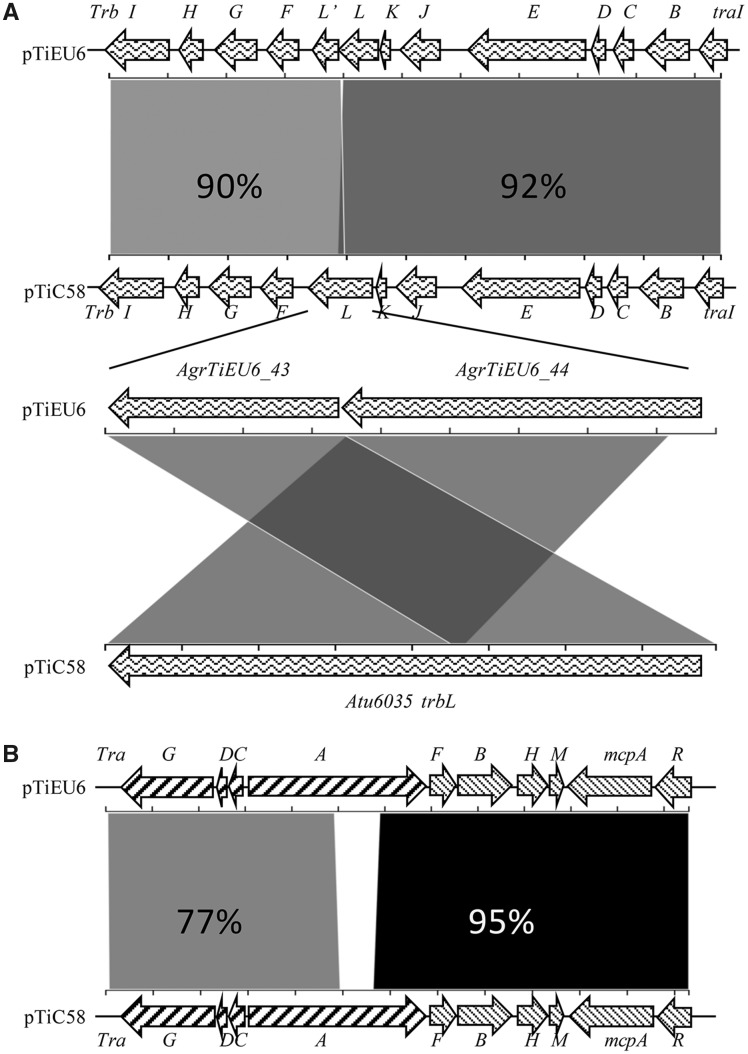
—Schematic comparison of the *tra* and *trb* operons of pTiEU6 with those of pTiC58. The alignment was calculated using the Blast 2.2.31+ algorithm (NCBI) at nucleotide level and visualized by the web-tool Kablammo (Wintersinger and Wasmuth, 2015). The arrows represent genes in pTiEU6 at the top and in pTiC58 at the bottom, respectively. The identity values of each single gene at amino acid level are present in supplementary table S2, Supplementary Material online.

Gene clusters associated with plasmid replication and maintenance (*rep*) and virulence (*vir*), share very strong similarities with those in all nopaline Ti-plasmids (∼99% identical at nucleotide level) (supplementary fig. S2, Supplementary Materials online). A similar strong similarity of 90–92% was seen for the *trb* operon encoding the type4 secretion system (T4SS) involved in conjugative plasmid transfer (fig. 5; supplementary table S2, Supplementary Materials online). It was remarkable that in contrast to the nopaline Ti-plasmids the *trbL* gene was split into two individual ORFs (AgrTiEU6_43 and AgrTiEU6_44) in reverse order in pTiEU6. Also the pTiEU6 plasmid has an almost identical set of *acc* genes involved in agrocinopine import and catabolism (99% identity with those of pTiC58 and pTiSAKURA at the nucleotide level). It suggests that the *rep*, *vir*, *acc*, and *trb* operons evolved together. Although the *tra* operons of pTiEU6, responsible for conjugative DNA processing of the Ti-plasmid, were almost identical to those of pTiSAKURA and pTiT37, the *traCDG* and *traA* operons shared only 77% similarities with those of pTiC58 (fig. 5; supplementary table S2, Supplementary Material online). Pairwise alignments of the *tra* operons of nopaline Ti-plasmids revealed that the *tra* operons of nopaline Ti-plasmids were closely related to each other, with the exception of those of pTiC58. This suggests that a recombination event took place in this area in pTiC58 or in an early descendant of pTiC58 before the diversification of the nopaline Ti-plasmids. The *tra* operons of pTiC58 are more related to those of pAtS4c (94% nucleotide identity) and the nopaline catabolic plasmid pAtK84b (88% nucleotide identity).

### T-Region

**Fig. 6.**
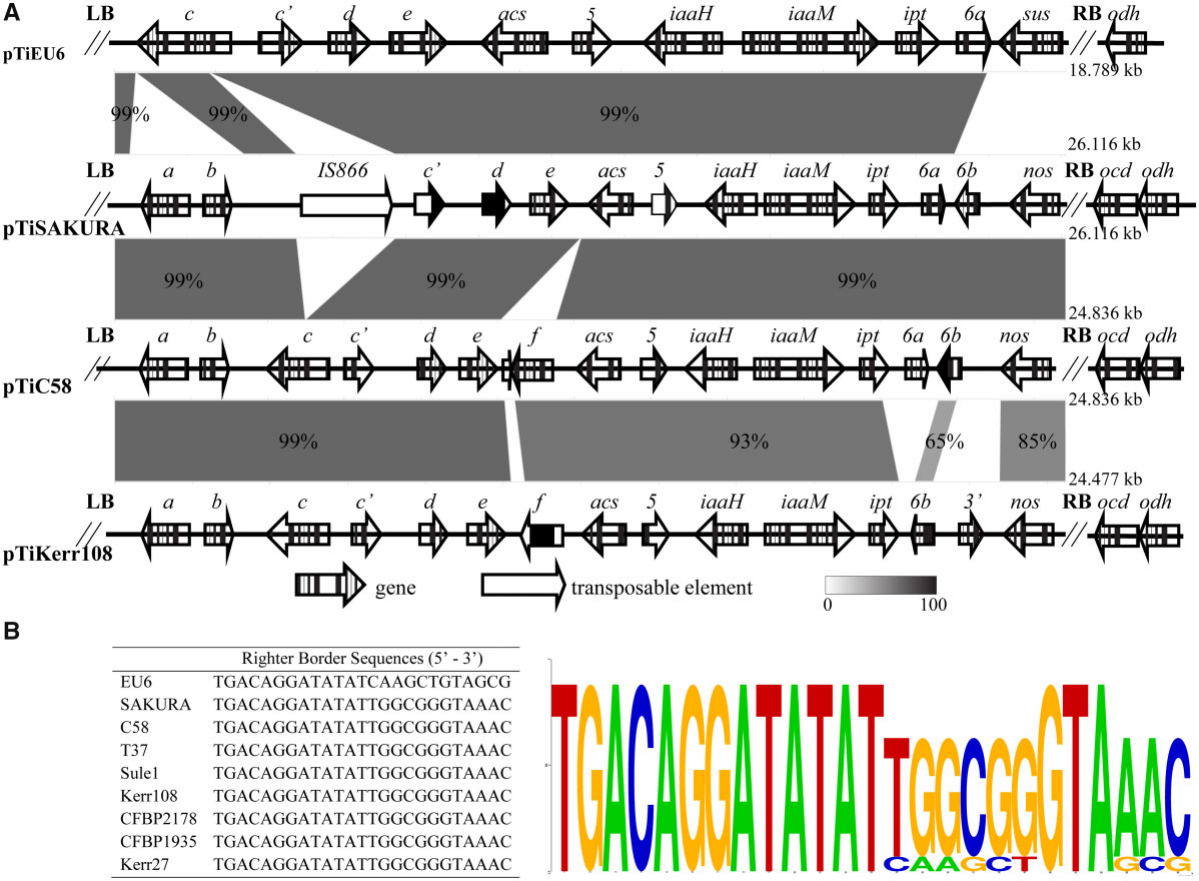
—(*A*) Structure of T-DNAs of pTiEU6 and nopaline pTiC58, pTiKerr108, and pTiSAKURA. Locations of open reading frames are shown by arrows. The pairwise alignments were calculated using the BlastN algorithm and visualized by the web-tool Kablammo (Wintersinger and Wasmuth, 2015). (*B*) Multiple sequence alignments of T-DNA right border repeats from pTiEU6 and nopaline Ti-plasmids were generated by WebLogo and a consensus is shown.

The T-region of pTiEU6 defined by conserved border repeats has a length of 18,789 bp (fig. 6*A*). Comparing the T-DNA of pTiEU6 to those of nopaline Ti-plasmids as first described by Otten et al. (1999) for pTiC58, it was found that the left border is identical to that of nopaline-type Ti-plasmids, but the right border is somewhat different from that of nopaline Ti-plasmids (fig. 6*B*). The T-DNA carries the essential oncogenes involved in the synthesis of the plant hormones auxin (*iaaH* and *iaaM*) and cytokinin (*ipt*). Also for the remaining part the T-region of pTiEU6 is collinear with those of the nopaline Ti-plasmids, including a gene for agrocinopine synthase (*acs*). As in the subgroup A nopaline Ti-plasmids (Broer et al. 1995) gene f is missing from the T-region of pTiEU6 (fig. 6*A*). In comparison to the nopaline T-region that of pTiEU6 is truncated at both ends. At the left a gene encoding a putative second agrocinopine synthase torf6/orf172 (*acs*) and gene 5 are absent and at the right end the area occupied by oncogene *6b* and the nopaline synthase gene is replaced by a novel gene (AgrTiEU6_18). Blundy et al. (1986) showed that the sequences at the right end of the T-region are involved in the biosynthesis of succinamopine. Gene AgrTiEU6_18 encodes a protein which contains a NAD/NADP dehydrogenase domain as is also present in nopaline synthase, but otherwise is very different. This gene must be responsible for succinamopine synthesis and we shall call AgrTiEU6_18 from now on *sus* as the gene for succinamopine synthase. It is important to remark here that *sus* is very different from the gene *les*, sometimes also called *sus* and responsible for succinamopine and leucinopine biosynthesis in tumors induced by agropine and chrysopine strains (Shao et al. 2018). This is not unexpected as succinamopine strains such as EU6 induce tumors with d^glu^,l^asp^-succinamopine, whereas agropine strains such as Bo542 and chrysopine strains such as Chry5 induce tumors with L^glu^, L^asp^-succinamopine (Chilton 1985a, 1985b). In order to avoid confusion we propose to call the previously described Bo542 and Chry5 genes encoding the enzyme catalyzing the production of the l,l-isomer *susL* and the EU6 gene encoding the enzyme catalyzing the formation of the d,l-isomer *susD*. The *susD* and *susL* genes are very different, but it is intriguing that they contain two small areas of similarity, one of which is present in the N-terminal part of the protein encoded by *susD*, but in the C-terminal part of the protein encoded by *susL* (supplementary fig. S5, Supplementary Materials online).

When comparing the T-regions of the nopaline Ti-plasmids, it was found that pTiKerr108, pTiCFBP1935, pTiCFBP2178, and pTiSule1 lack gene *6a* and instead have another gene called *3*′ inserted between gene *6b* and the *nos* gene (fig. 6*A*). Although a *6b* gene is still present, this exhibited only a low nucleotide similarity of 65% with the gene *6b* encoded by the other nopaline Ti-plasmids in this study (supplementary fig. S3, Supplementary Materials online). The gene *3*′ showed 71% nucleotide similarity with the corresponding gene of octopine-type Ti-plasmids. This suggests that genes 6a and 6b were replaced by gene 3′ and a different version of 6b by a local DNA recombination event with another Ti-plasmid. These differences with pTiC58 were noted before by the Otten lab, after sequencing this part of the T-region of the nopaline Ti plasmid pTi82.139, which is identical to pTiCFBP2178 (Drevet et al. 1994; Otten and De Ruffray 1994).

### Succinamopine Catabolism

Nopaline Ti-plasmids have strongly conserved genes involved in nopaline import and catabolism (*noc*), which are located adjacent to the right border of the T-region. In pTiEU6 these genes are absent, but replaced by another set of genes. Tumors induced by strains containing pTiEU6 do not contain nopaline, but instead succinamopine/asparaginopine is present, which is related to nopaline, but in which arginine is replaced by asparagine (Chang and Chen 1983; Chilton et al. 1984). Besides succinamopine (SAP), succinamopine lactam (SAL), and succinopine lactam (SOL) were found in these tumors (Chilton et al. 1984). The set of genes adjacent to the RB of the T-region (AgrTiEU6_20–AgrTiEU6_28) shares significant homology with the genes involved in succinamopine/leucinopine import of agropine plasmid pTiBo542 and chrysopine plasmid pTiChry5 (table 2). Directly adjacent to the RB are three genes (AgrTiEU6_20–AgrTiEU6_22, SacBCD) for an ABC transport system, which components have 79%, 87%, and 82% similarities at amino acid level with the components LecBCD of the succinamopine/leucinopine import system from agropine pTiBo542 and chrysopine pTiChry5, but with undetectable or only low similarity with those of the nopaline import system. A gene encoding a periplasmic substrate binding protein can be found close by (AgrTiEU6_28, SacA), which shows significant similarity to the leucinopine/succinamopine binding proteins from pTiBo542 and pTiChry5 (LecA, 70% similarity at amino acid level), but which has much less similarity to the corresponding nopaline binding protein NocT. Although it was thought that flavin-containing opine NAD(P)H-dependent dehydrogenase activities were encoded by two genes in the nopaline Ti plasmid called *noxA* and *noxB* (Zanker et al. 1994), recently evidence was provided that in fact such activity requires a small third gene called *noxC*, which provides an [2Fe–2S] iron-sulfur cluster to the enzyme (Watanabe et al. 2015). The enzymes encoded by genes AgrTiEU6_24/25/26 (*sacGFE*) show ∼40% similarity with NoxACB at amino acid level*.* Remarkably, a fourth gene is present AgrTiEU6_23 (*sacH*) which encodes a protein which shows much less similarity with that encoded by *noxB*, but still has a similar architecture as NoxB and which shows ∼50% similarity with the proteins encoded by the *lecH* genes from pTiBo542 and pTiChry5 at amino acid level. The *lecH* gene together with genes *lecG* and *lecF* encodes the dehydrogenase necessary for leucinopine/succinamopine catabolism. Both in AgrTiEU6_23 (*sacH*) and in AgrTiEU6_26 (*sacE*) the signature for FAD-binding can be seen (supplementary fig. S4, Supplementary Materials online). Such a cluster of four genes with two “*noxB*” components was previously found in *Bradyrhizobium japonicum*, where it was shown that two different ABC complexes can be formed, both with catalytic activity (Watanabe et al. 2015). It is likely, therefore, that also in strains carrying the pTiEU6 plasmid two different complexes are formed for the catabolism of different opines including succinamopine and leucinopine.

**Table 2:** Relatedness among the Predicted Gene Products of pTiEU6 Opine Catabolism Region and Those of Other Ti-Plasmids

locus_tag	Gene	pTiC58	pTiBo542	pTiChry5	pTiAch5
AgrTiEU6_20	*sacD*	*nocP 33%*	*lecD 79%*	*lecD 80%*	*—*
AgrTiEU6_21	*sacC*	—	*lecC 87%*	*lecC 87%*	*—*
AgrTiEU6_22	*sacB*	*—*	*lecB 82%*	*lecB 82%*	*—*
AgrTiEU6_23	*sacH*	*—*	*lecH 50%*	*lecH 49%*	*—*
AgrTiEU6_24	*sacG*	*noxA 42%*	*lecG 47%*	*lecG 48%*	*ooxA 39%*
AgrTiEU6_25	*sacF*	*—*	*lecF 54%*	*lecF 56%*	*—*
AgrTiEU6_26	*sacE*	*noxB 43%*	*—*	*—*	*ooxB 41%*
AgrTiEU6_27	*sacR*	*nocR 33%*	*lecR 59%*	*lecR 61%*	*—*
AgrTiEU6_28	*sacA*	*—*	*lecA 70%*	*lecA 70%*	*—*

Note.—Values of relatedness are percentage of identities for the pairs of amino acid sequences. The dash means no similarities.

Directly adjacent to the RB of pTiEU6 gene AgrTiEU6_19 is present, predicted to encode an NAD-dependent opine dehydrogenase (*odh*). The ODH encoded by AgrTiEU6_19 shows strong similarity to the ODH encoded by nopaline Ti plasmids (64% similarity with that of pTiC58 at amino acid level). Its role in opine catabolism has not been clarified as yet. Nopaline catabolism liberates α-ketoglutaric acid and arginine and the nopaline Ti plasmid has genes encoded on the Ti plasmid for arginine and ornithine catabolism. These genes are absent from pTiEU6, which has a gene encoding an asparaginase instead (AgrTiEU6_32), which can cope with the asparagine liberated when succinamopine is split into asparagine and α-ketoglutaric acid. Genes (AgrTiEU6_30 and 31) encoding a couple of proteins with the signature of hydantoinase/oxoprolinase A and B are present adjacent to the gene encoding asparaginase. Such genes are also present in the nopaline catabolism area of the nopaline Ti-plasmids. These proteins could open lactam rings and may therefore be necessary for the catabolism of the lactam opines SAL and SOL in the case of pTiEU6. A LysR type transcriptional regulator (AgrTiEU6_27) is present within the area and this may control expression of the succinamopine import and catabolism genes. Finally this region of the Ti plasmid contains another set of genes for an ABC transporter (AgrTiEU6_34–AgrTiEU6_37), most related to putrescine (*potABCD*) transport systems, which is accompanied by a gene for a second LysR type transcriptional regulator (AgrTiEU6_33), which shares 30% similarity with AgrTiEU6_27. Finally this segment of the Ti-plasmid ends with a gene *mmgE* (AgrTiEU6_38), encoding an enzyme with similarity to 2-methylcitrate dehydratase, which is necessary for catabolism of (potentially toxic) propionyl-CoA propionate, which may be formed when catabolizing leucinopine.

## Discussion

Traditionally, Ti-plasmids are classified on the basis of the opines that are generated in the infected tumors. In this study we reported the first complete sequence of a succinamopine-type Ti-plasmid pTiEU6 and its close relatedness to nopaline Ti-plasmids. Six nopaline Ti-plasmids from strains from different geographical origins were newly sequenced in order to trace the evolutionary history of nopaline and succinamopine Ti-plasmids.

The succinamopine Ti-plasmid pTiEU6 shares with nopaline Ti-plasmids a number of highly conserved regions including most of T-region, replication, conjugation, and virulence region. Based on whole plasmid sequence comparison, pTiEU6 is most similar to pTiT37 and pTiSAKURA, which form a subgroup A of nopaline Ti-plasmids. They only differ considerably in the region related to the biosynthesis and catabolism of succinamopine or nopaline, even though the structures of these opines are similar. The area between *rep* and *tra* operon is conserved in pTiEU6 and subgroup A, but is different from that of the subgroup B nopaline Ti-plasmids. Moreover, pTiEU6 and subgroup A nopaline Ti-plasmids carry the same transposable elements, including a diagnostic insertion of IS1182, which is absent from subgroup B nopaline Ti-plasmids. Finally, from the T-region of the subgroup A nopaline Ti-plasmids and pTiEU6 gene f is deleted. Taken together, it can be assumed that pTiEU6 is derived from a common ancestor with subgroup A nopaline Ti-plasmids (pTiT37 and pTiSAKURA) in which the opine catabolic region and the right part of the T-region was replaced by that of an unknown succinamopine plasmid by DNA recombination.

By comparison of the complete Ti-plasmid sequences, the nopaline Ti-pasmids can be classified into two subgroups, which we have called A and B and which differ in an area located between the *rep* and *tra* genes. The insertion of transposable elements can be used to follow the evolutionary path. Although the nopaline Ti-plasmids studied all contain (the remnants of) three transposable elements (IS21, IS110, and ISRel8) at an identical site, only the subgroup A plasmids specifically contain a copy of IS1182, whereas only the subgroup B plasmids carry a copy of IS692 near ISRel8. Interesting, plasmid pTiC58 lacks both of them. In general, major evolutionary changes only take place once. We therefore suggest that pTiC58 represents the most ancestral of the nopaline Ti-plasmids in our study. It has all conserved sectors, but lacks both transposable elements IS1182 and IS692. The insertion event of IS1182 defines subgroup A, which has kept the *6a* and *6b* genes as in C58, but has undergone a major exchange (recombination event R1; fig. 4*B*) with another plasmid in the segment between replication *rep* and conjugation *tra* operons. One or more further recombination events R2 (fig. 4*B*) led to exchanges of the right part of the T-region and the adjacent catabolic region and resulted in the formation of the succinamopine Ti-plasmid. The acquirement of IS692 marks the evolution of subgroup B. Acquisition of IS692 was followed by a putative DNA recombination event R3 (fig. 4*B*) with another Ti-plasmid leading to a replacement of the area occupying genes 6a and 6b with a new segment containing a very different gene 6b and gene *3*′. The result of this event can be seen in all the subgroup B plasmids except pTiKerr27, which has otherwise undergone severe changes at three positions probably coinciding with transposition events. Our data suggest that pTiC58 is the most closely related to the ancestral nopaline Ti-plasmid. By a putative recombination event R4 part of the *tra* region of pTiC58 is deviant from that of the other nopaline Ti-plasmids. During the preparation of our manuscript, the characterization of the closely related nopaline Ti-plasmids pTiC5.7/pTiC6.5, which are almost identical to plasmid pTiKerr108 from our study, was reported and the relatedness of nopaline Ti-plasmids to nopaline catabolic plasmids was determined leading to the hypothesis that the nopaline Ti-plasmids are derived from such catabolic plasmids (Kuzmanović and Puławska 2019). Interestingly, the *tra* genes of the pTiC58 are more similar to those of nopaline catabolic plasmid pAtK84b (88% nucleotide identity) than to those of any of the other nopaline Ti-plasmids in our study, which would be in line with pTiC58 being the most ancestral of the studied plasmids and recombination event R4 occurring after formation of the ancestral nopaline Ti-plasmid, but before the transposition events leading to subgroups A and B.

## Conclusion

The sequence of the succinamopine Ti plasmid confirms its close relatedness to the nopaline Ti plasmids. By a detailed comparison to the full sequences of the two published and 6 newly sequenced nopaline Ti-plasmids we can infer that the succinamopine Ti plasmid is evolutionary derived from the group A nopaline Ti plasmids (pTiSAKURA, pTiT37), which is characterized by the presence of a copy of IS1182 at a specific location and a recombination event leading to a new set of genes between the *tra* genes and the *rep* genes in comparison to other nopaline Ti-plasmids. In pTiEU6 the area occupying the right part of the T-region (including gene *6b* and *nos*) and the cluster of genes involved in nopaline import and catabolism up to the conserved *trb* cluster involved in conjugation was replaced by a new set of genes that is overall more similar to that present in chrysopine and agropine Ti plasmids and involved in succinamopine/leucinopine synthesis, import and catabolism. It is likely that this was brought about by a recombination event between a group A nopaline Ti plasmid and another type of Ti plasmid. Precedent for recombination events between Ti plasmids has been seen in in vitro experiments, when introducing octopine Ti plasmids into nopaline strains (Hooykaas et al. 1980). Initially this leads to co-integrate formation, but eventually such co-integrates may disintegrate leading to new types of (Ti) plasmids.

## Supplementary Material

Supplementary data are available at *Genome Biology and Evolution* online. 

## Supplementary Material

evz173_Supplementary_Data

## Notes

**Data deposition:** The complete nucleotide sequences of the Ti-plasmids sequenced in this study have been deposited at GenBank under the following accession numbers: pTiEU6, KX388535; pTiSule1, MK439381; pTiCFBP2178, MK439382; pTiCFBP1935, MK439383; pTiKerr108, MK439384; pTiKerr27, MK439385, and pTiT37, MK439386.
