# Supplementary material for: Complete Sequence of Succinamopine Ti-Plasmid pTiEU6 Reveals Its Evolutionary Relatedness with Nopaline-Type Ti-Plasmids
Source: Genome Biol Evol. 2019 Aug 6;11(9):2480–91. doi: 10.1093/gbe/evz173 (PMC6733357; doi:10.1093/gbe/evz173)
Supplement: evz173_Supplementary_Data [file evz173_supplementary_data.zip › Supporting Information Legends.docx]

**Supplementary Materials**

**Figure S1** A survey on the distribution of *Garfield* in some insects using PCR. All PCR products were confirmed by cloning and sequencing. “M” represents the molecular marker. 1. *Bombyx mori*; 2. *Rhodnius prolixus*; 3. *Antheraea yamamai*; 4. *Antheraea pernyi*; 5. *Samia luzonica*; 6. *S. insularis*; 7. *Amathuxidia amythaon*; 8. *Caligo eurilochus*; 9. *S. cynthia ricini*.

**Figure S2** Multiple alignments of *Garfield* obtained by cloning and sequencing. Species abbreviations: SL, *Samia luzonica*; SC, *S. cynthia ricini*; BM, *Bombyx mori*; RP, *Rhodnius prolixus*; AP, *Antheraea pernyi*. The poly (T) located at the 3′ end of the insertion was indicated using black line rectangle, and their potential TSD was showed using arrows in the same direction.

**Figure S3** Alignment between homologous regions of the insertion found in *Garfield* element and two *Zenon* sequences: A) 5’ end, and B) 3’ end. The dashed red line indicate alignment obtained through the Censor search in Repbase Update database.

**Figure S4** Phylogenetic analysis of Zenon elements based on the reverse transcriptase domain. Symbols at nodes represent support based on Maximum Likelihood bootstrap/Bayesian posterior probability, as reported on the upper left legend. Each element has been labelled by a suffix indicating the pertaining species, as reported in Figure 2 legend.

**Figure S5** Comparison of *Persaeus*/*Zenon* phylogenetic trees within the *Antheraea* (A), *Papilio* (B) and *Spodoptera* (C) clade with those of their hosts. Dashed lines connect *Persaeus* elements with the respective species; red lines indicate phylogenetically discordant/conflicting positions. Symbols at nodes represent support based on maximum likelihood bootstrap/Bayesian posterior probability, as reported on the top legend. Each element has been labelled by a suffix indicating the pertaining species, as reported in Figure 2 legend. Host species phylogenies redrawn after (Singh et al. 2017; Zakharov et al. 2004; Kergoat et al. 2012).

**Table S1** Genome occupancy of *Persaeus* and *Zenon*.

**Table S2** Percentage of similarity at homologous regions between *Persaeus* and *Zenon* pairs.

**Dataset S1** Multiple alignments of consensus sequences of all *Persaeus* and *Zenon* identified in this study.
