# Supplementary figures and images for: Complete Sequence of Succinamopine Ti-Plasmid pTiEU6 Reveals Its Evolutionary Relatedness with Nopaline-Type Ti-Plasmids
Source: Genome Biol Evol. 2019 Aug 6;11(9):2480–91. doi: 10.1093/gbe/evz173 (PMC6733357; doi:10.1093/gbe/evz173)

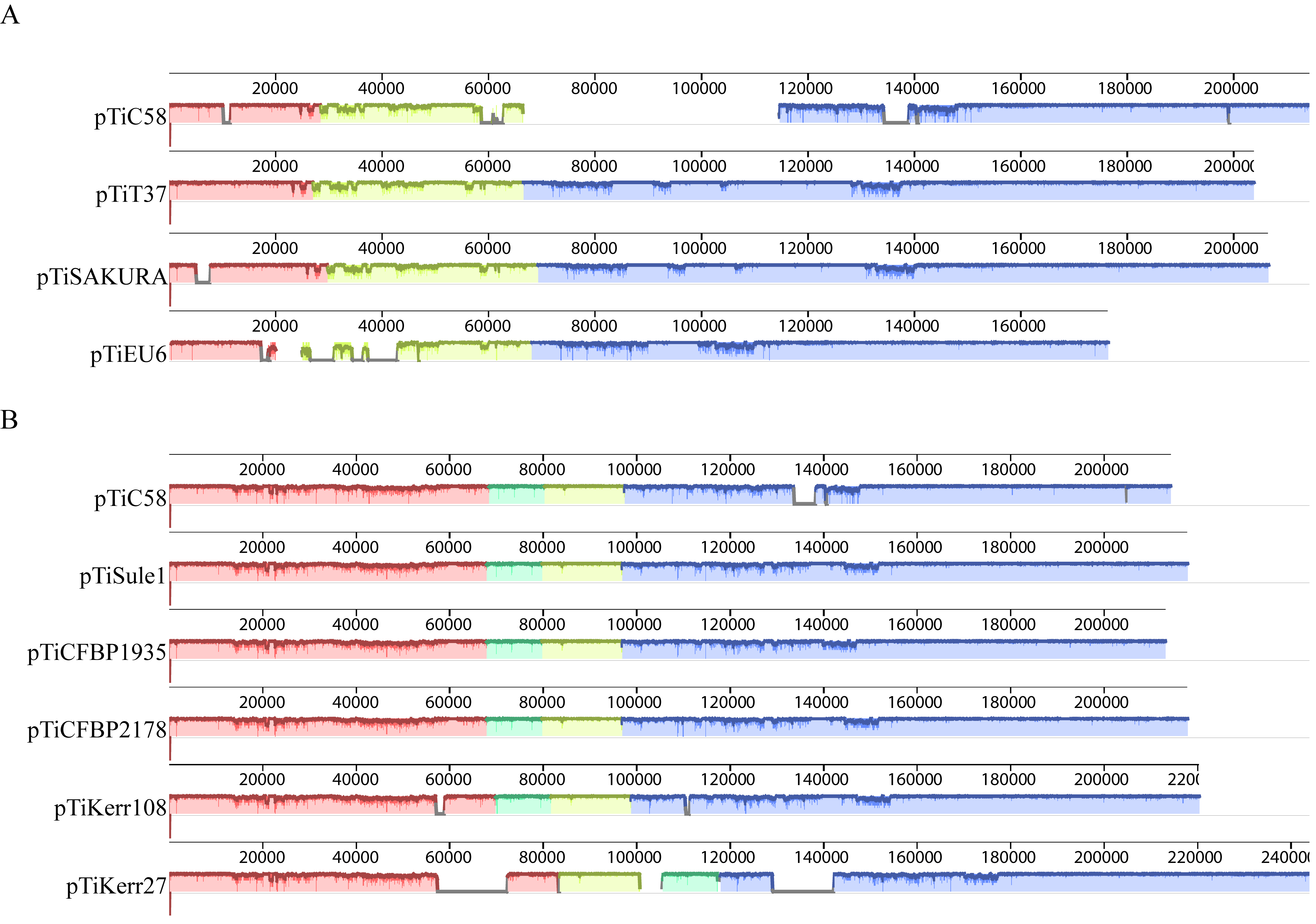

Supplement: evz173_Supplementary_Data [file evz173_supplementary_data.zip › Figure S1 subgroup A and B comparison.tif]

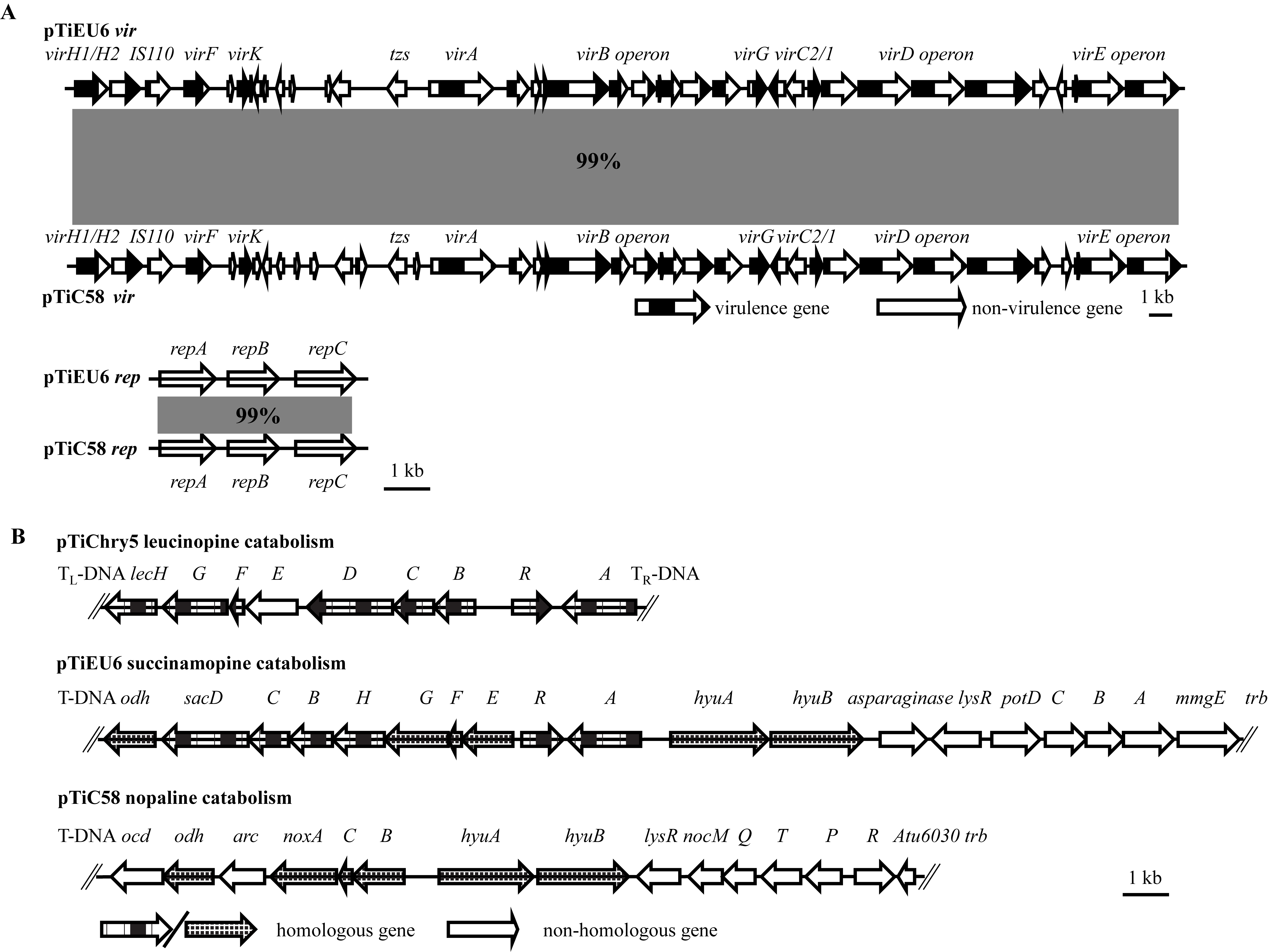

Supplement: evz173_Supplementary_Data [file evz173_supplementary_data.zip › Figure S2new vir, rep , sac gene cluster.tif]

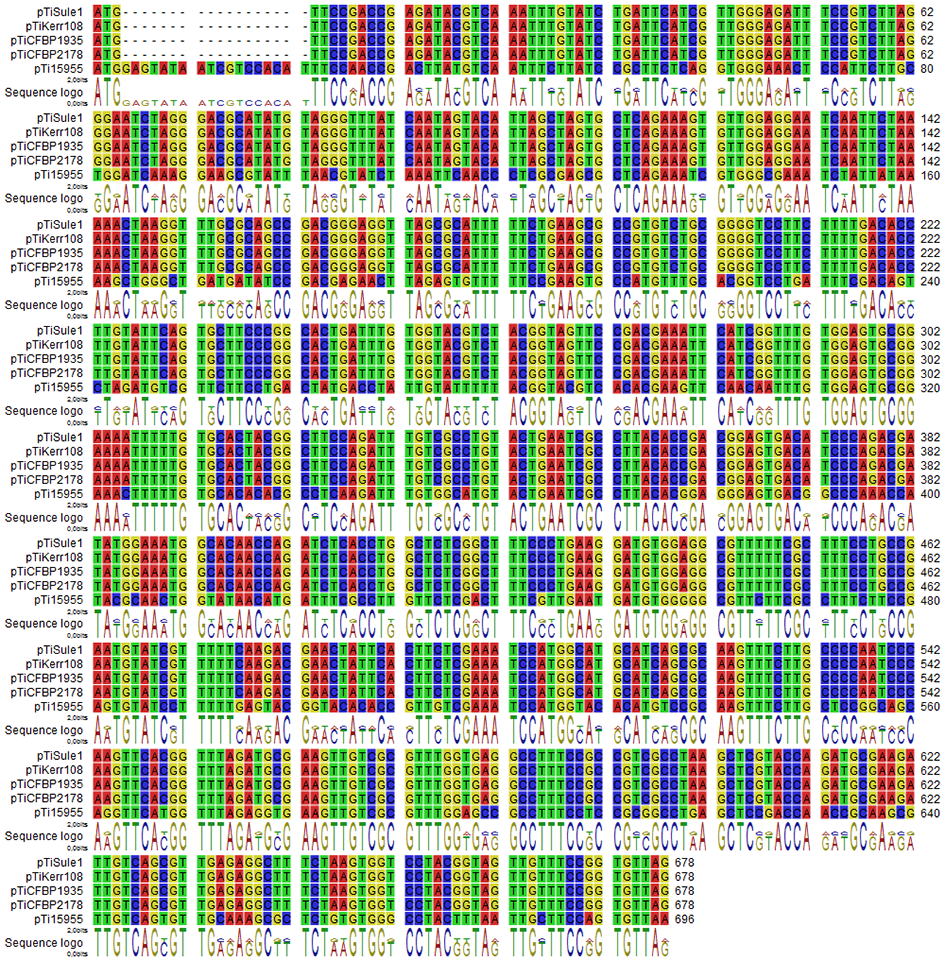

Supplement: evz173_Supplementary_Data [file evz173_supplementary_data.zip › Figure S3A 3' gene.tif]

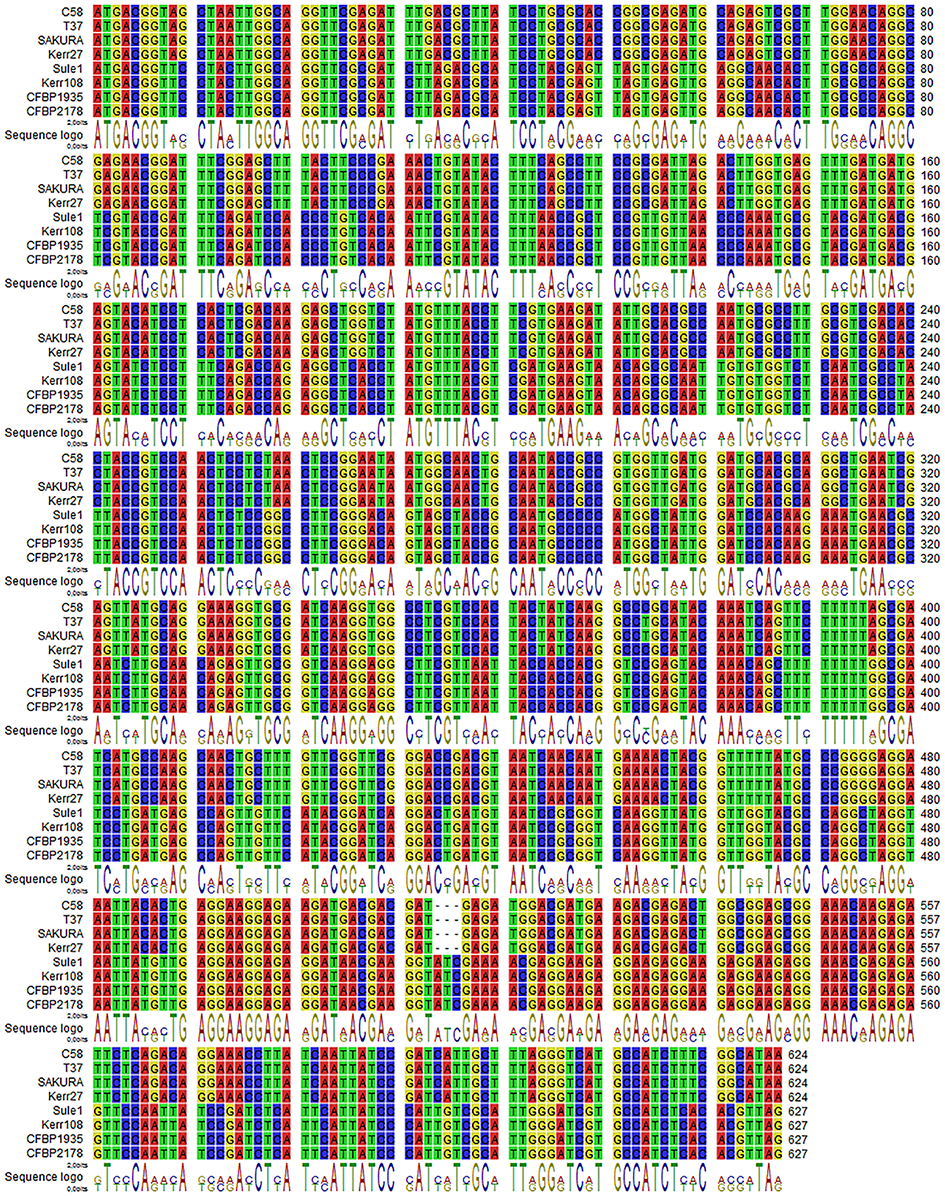

Supplement: evz173_Supplementary_Data [file evz173_supplementary_data.zip › Figure S3B 6b gene.tif]

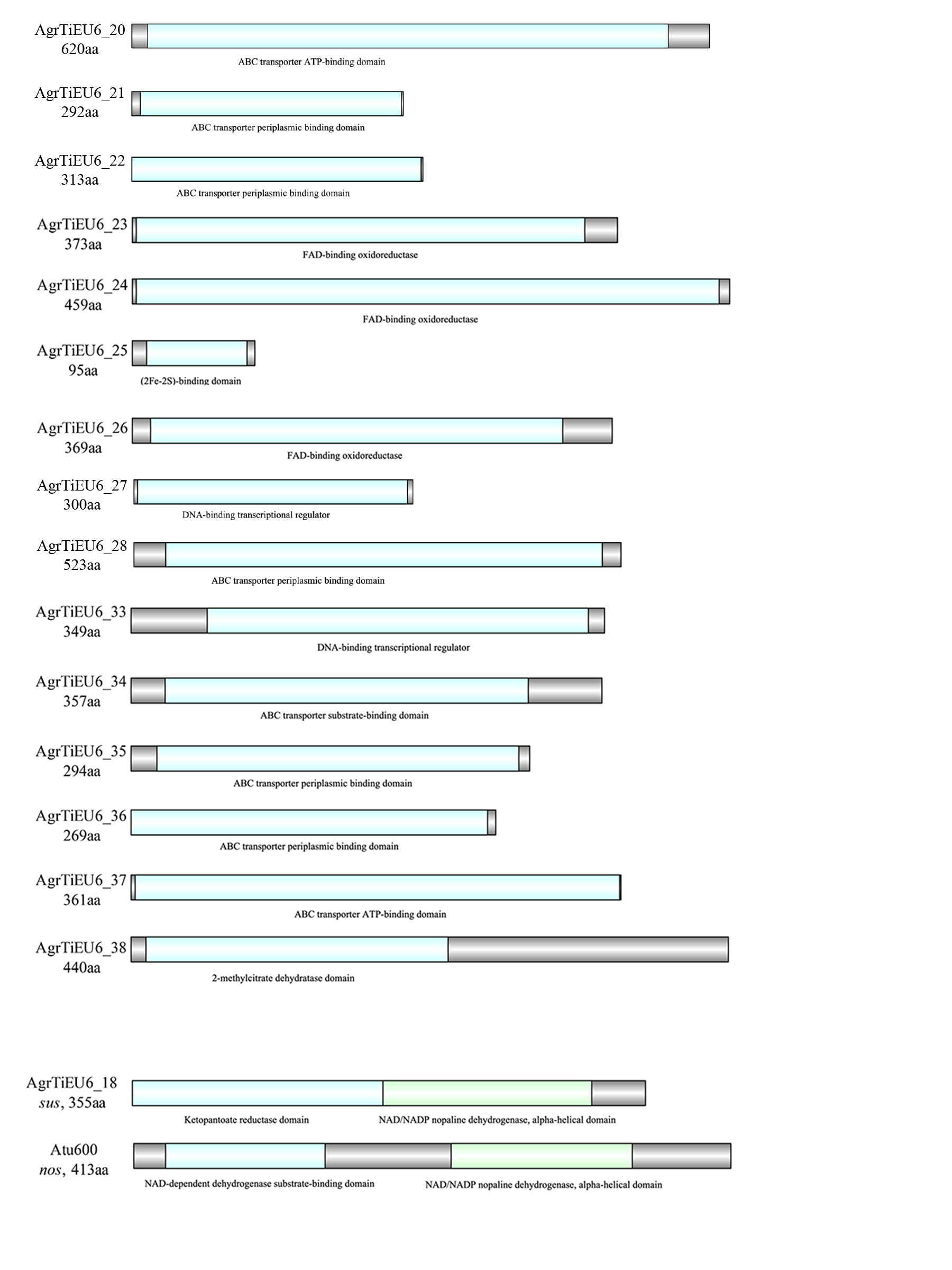

Supplement: evz173_Supplementary_Data [file evz173_supplementary_data.zip › Figure S4 Domain structures of opine-related proteins.tif]

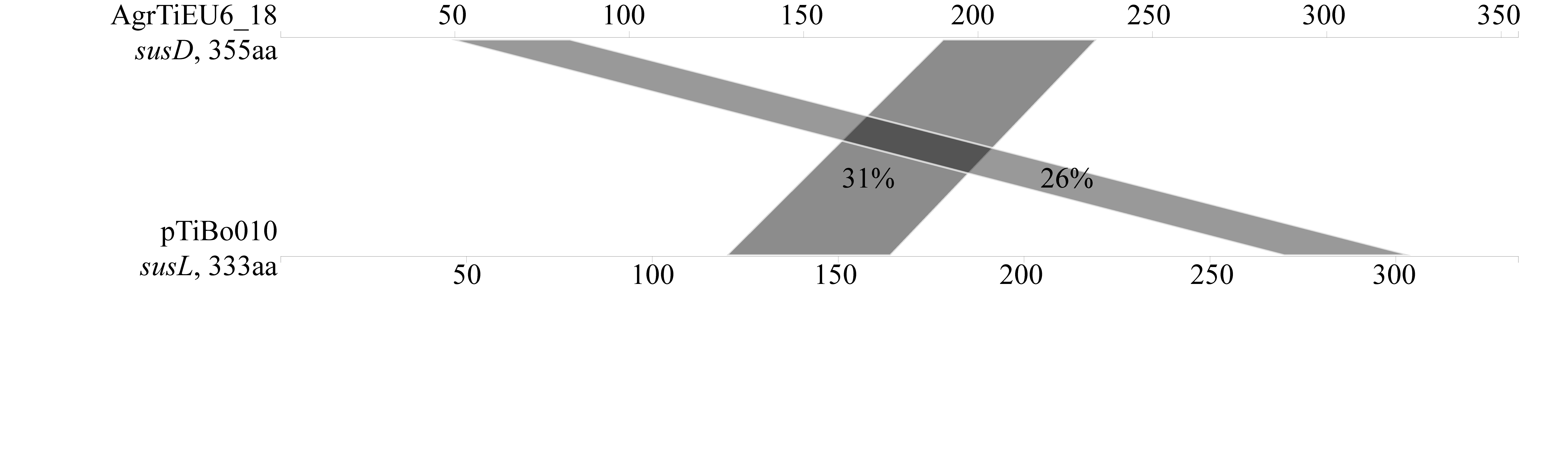

Supplement: evz173_Supplementary_Data [file evz173_supplementary_data.zip › Figure S5 Comparison of susD and susL.tif]
